# Event and Comment

**Published:** 1909-02-15

**Authors:** 


### DENTAL REGISTER.

**Vol. LXIII. February 15, 1909. No. 2.**

### EVENT AND COMMENT.

INSTITUTE OF DENTAL PEDAGOGICS. The sixteenth annual meeting of this most interesting society met in St. Louis, December 30th and 31, and January 1st. The prediction ten years ago that this society would be short-lived. seems far from being true, for at this meeting over thirty colleges were represented, and delegates came from Boston, New York, San Francisco, New Orleans and Minneapolis, about as remote distances from the place of meeting as one could expect delegates to come for a meeting from which they would be unable to get any great benefit. But there was no sign of senility or lack of enthusiasm. The three days were crowded full of intensely interesting material and it was with the greatest embarrassment that the presiding officer was able to stop the discussions when the time had expired. The only criticism we heard was that there wasn't time enough to discuss the topics presented as much as was desirable. The meetings were held in the Planters Hotel, where the arrangements were very good, indeed. Three sessions each day were held, except the last day, and many excellent papers were read and well discussed. The program was one of the best ever presented and it was not interrupted by social functions or junketing parties. The exhibits were not many, but most of them exceedingly interesting and of great value. The exhibits are not so important items as in the beginning of this organization, when the idea of the promoters was a technic discussion only. Now the papers take a much broader pedagogic view, and some of the most interesting themes of discussion were on the teaching of scientific subjects. This proves that the vigorous contest of a few in the earlier years of the organization to widen the plan of the association was most wise and providential. The Institute will meet next year at Toronto, with the Royal College of Dental Surgery of Ontario. It is anticipated that this will be a most interesting meeting, as our Canadian friends are determined to show us Canadian progress and hospitality.

**DENTAL JOURNALISM.** If we are to judge by the evidences of change and improvement in form and make-up of our exchanges, the year 1909 will see decided improvement in our periodic literature. The *Dental Summary* has come out in new dress and enlarged size; The *Dental Era* promises to do a similar stunt; The *Dental Digest* has passed to the control of the Twentieth Century Supply Company, with that indefatigable worker Dr. George Clapp as chief pusher; The *Journal of the Allied Societies* comes to us double size and full of beautifully illustrated articles of great merit and originality; The *Dentists Magazine* has lost its aggressive and able quintette of editors, but we hear it will be succceeded by another of equal ability and aggressiveness. The older journals have made no special manifestations of new birth, but we can depend on them to line up in their usual form and to deliver their share of the good things of the year. It really seems that our profession is coming to its senses in the matter of its literature, and that we are reading more than ever before in the profession's history. We are writing and reading papers and text books as never before; and it is not because our *trade houses* are trying to out-do each other in presenting attractive advertisement of their wares to us in dollar journals of seductive designs and appearance, that we are increasing in appreciation of our literature. As proof that we are really appreciative of our literature may be cited the fact that there never was a time when books were bought so largely nor was there ever so active an interest in their preservation in private as well as public libraries. Let us hope that our dental societies may increase in membership, our journals vie with each other in reporting their proceedings in detail, and our publishers may not relax in their efforts to give us high class periodicals in form as well as matter.

